# Evolution of the Conceptualization of Filial Piety in the Global Context: From Skin to Skeleton

**DOI:** 10.3389/fpsyg.2021.570547

**Published:** 2021-03-29

**Authors:** Olwen Bedford, Kuang-Hui Yeh

**Affiliations:** ^1^Department of Psychology, National Taiwan University, Taipei, Taiwan; ^2^Institute of Ethnology, Academia Sinica, Taipei, Taiwan

**Keywords:** filial piety, intergenerational relations, familism, relationalism, dual filial piety model, motivations, authoritarian parenting, elder care

## Abstract

Social science researchers often define *filial piety* as a set of norms, values, and practices regarding how children should behave toward their parents. In this article, we trace the conceptual development of filial piety research in Chinese and other societies to highlight the assumptions underlying this traditional approach to filial piety research. We identify the limitations of these assumptions, including the problem of an evolving definition and lack of cross-cultural applicability. We then advocate an alternative framework that overcomes these limitations by focusing on the deep structure of filial piety: the dual filial piety model (DFPM). The DFPM applies the concept of contextualized personality to reconceptualize filial piety in terms of authoritarian and reciprocal psychological motivations particular to the parent-child context. Because the focus is on a universal psychological mechanism rather than cultural norms, values, and behavior, the DFPM may be applied for investigation of filial piety at individual, social, and cultural levels within and across various societies. We discuss application of the DFPM in relation to existing filial piety and intergenerational relations research from several societies and conclude with a comparison to other recent proposals for measuring Chinese filial piety.

## Introduction

The call for contributions for the collection of articles in this special topic defined *filial piety* as “a set of norms, values and practices regarding how children should behave toward their parents,” and noted that filial piety is important “for cultures where a family is regarded as an important basic unit of social relationships.” The focal concern is that “psychologists do not seem to have many conclusions over what defines filial piety, how it is assessed, and its impact on different relationships in the distinctive socio-cultural contexts.”

The perspective embedded in these statements specifies how filial piety is conceptualized and the cultures in which it is expected to be most relevant. This perspective underlies the majority of filial piety research. We assert that it is this very conceptualization that has led to the current situation in which there seems to be little consensus on the definition, assessment, and impact of filial piety. The lack of agreement on how to best conceptualize and measure filial piety also makes it difficult for scholars to build on each other’s work, which may in turn impede the progress of filial piety research. Scholars must share a common starting point in order to advance knowledge about filial piety.

In the present article, we review the evolution of the traditional conceptualization, interrogate the assumptions underlying it, and highlight its limitations. Our purpose is to advocate an alternative framework that provides a basis for a definition of filial piety that functions across socio-cultural contexts and is thus appropriate for investigation within and across various societies.

We ground our discussion in an examination of the early studies of filial piety in Chinese societies as well as research on filial concepts in other societies in order to demonstrate the challenges with the current conceptualization, which primarily focuses on surface behavior and attitudes to norms. Understanding how the early literature conceptualized filial piety is important because the authors of more recent studies draw on it to justify their definition of filial piety and they employ items from the older measures to create new measures (e.g., Lee and Kwok, 2005; Cheng and Chan, 2006; Lum et al., 2016; Fu et al., 2020). We propose a paradigm shift from the current focus on surface-level analysis of behavior and attitudes to a deep structure analysis of the motivations supporting this behavior. We highlight a recent deep structure approach grounded in relationalism in which filial piety is viewed as a contextualized personality construct and expressed in terms of authoritarian and reciprocal motivations: the dual filial piety model (DFPM). This approach allows investigation of a broader range of filial interaction and thus a broader range of filial outcomes because deep motivations can be used to explain surface behavior, but surface behavior does not in itself indicate motivation. We discuss application of the DFPM approach in relation to filial piety research from some non-Confucian societies to demonstrate the cross-cultural applicability of the model, and then conclude with a review of recent proposals for measuring Chinese filial piety (i.e., Lum et al., 2016; Shi and Wang, 2019; Fu et al., 2020).

## Early Psychological Conceptualization and Measurement of Confucian Filial Piety

The conceptualization of filial piety in the psychology literature as *a set of norms, values and practices regarding how children should behave toward their parents* began with the earliest psychology studies on this topic several decades ago (e.g., Ho and Lee, 1974). As scholars in Confucian societies noticed the trend toward smaller families, growing presence of women in the workplace, and increasing geographic mobility of workers, they began to debate the impact of societal modernization on intergenerational relationships and individual psychological well-being (Sung, 1995). Some scholars even questioned whether filial piety could survive Chinese communism because the central government had passed legislation to undermine it (Ikels, 2004). Ho and Kang (1984), p. 1004 described the debate as follows:

There are those who see a basic continuity with the past; that is, despite changes that have taken place, the essential “Chinese” character of child-rearing patterns among contemporary Chinese remains highly discernible. …[O]thers point to dramatic social changes that have taken place. They question, therefore, how meaningful it is to continue to speak of the “Chinese” pattern.

Early psychology researchers focused on measuring traditional filial beliefs and attitudes in order to investigate whether they were weakening with the modernization of Chinese societies. For example, Ho and Kang (1984) found that fathers depart more from the traditional orientation to filial piety than grandfathers. They concluded that intergenerational differences in filial piety scores reflect changes in traditional values and that “filial piety no longer commands the same degree of absolute observance as it once did” (Ho and Kang, 1984, p. 1010). In other words, they equated reduced endorsement of traditional norms with reduced observance of filial piety.

### Filial Piety as Traditional Norms

This conceptualization casts filial piety as a static set of beliefs or attitudes and practices anchored in traditional norms. Accordingly, Ho (1996) described a relationship between filial piety and cognitive conservatism, which Zhang and Bond (1998) claimed explained the significant negative correlation of filial piety with the personality trait of Openness. Zhang and Bond also identified a significant positive correlation with Neuroticism, which they postulated indicated that modern Chinese people exposed to the Western ideologies of freedom and independence would feel “emotional disturbances” if they also endorsed filial piety (p. 415). Other researchers echoed this concern, pointing out that filial piety beliefs may inhibit a person’s independence, or suppress creativity (e.g., Liu and Lin, 1988), and that people endorsing filial piety tended to have lower verbal fluency, and to be passive, uncritical, uncreative, fatalistic, superstitious, authoritarian, dogmatic, and conformist (Ho, 1994).

Evidence suggested that these personality characteristics carried over into parenting attitudes. For example, Ho (1996) found that filial piety beliefs corresponded to parenting attitudes with an emphasis on obedience, indebtedness, impulse control, and moral correctness, but not those characterized by self-fulfillment, self-expression, and psychological sensitivity. Parental filial piety beliefs also corresponded to higher rigidity and lower cognitive complexity in their children. Ho concluded that endorsement of filial piety beliefs “appears to be predominantly and consistently negative from a contemporary psychological perspective on human development” (1994, p. 362).

These types of studies focused on hierarchical authority ranking in the family, cognitive conservatism, and the tendency to judge people against moral standards. Ho and his colleagues viewed filial piety as a product of normative socialization. Scholars with this point of view concluded that filial piety is diminishing with modernization because “the social foundations for filial piety have been greatly undermined in Chinese society” (Ng, 1998, p. 101).

### Expanding the Conceptualization of Traditional Norms

Rather than viewing filial piety as a static set of norms that people may be moving away from, other scholars began with a different premise, which resulted in the opposite conclusion: filial piety is not diminishing in modern society. For example, Yang (1988) defined filial piety in terms of an attitude: *paramount concern for the well-being of one’s parents*. Starting from this perspective, Cheung et al. (1994) explored the relation between filial piety and family cohesion. They pointed out that the Confucian Book of Rites state that if caring for one’s parents is experienced as a burden, then the act does not qualify as filial piety. In other words, if children care for their elderly parents because they perceive it to be a requirement of an imposed norm, their actions do not count as filial piety. Cheung et al. concluded that filial piety is thus based on the development of empathy (i.e., an affective concept), not normative socialization or rational choice. They also suggested that normative characteristics of filial piety may be diminishing due to modernization, which may be causing affective aspects of filial piety to play a larger role in family cohesion.

Sung’s (1990; 1995) approach to conceptualizing filial piety entailed investigating how modern people perceive and enact it. He identified the ideals highlighted in contemporary experiences by analyzing interviews with people who had received national awards for demonstration of filial piety. His analysis identified two orthogonal factors. The first related to filial behavior and included sacrifice, responsibility, and repayment. The second related to filial emotion and encompassed love, family harmony, and respect. Sung (1995) found that the values supported by filial piety facilitate intergenerational relationships by supporting warmth, love, harmony, and close family ties. Participants most valued love and affection. In Sung’s view, the values underlying filial piety had not changed, just the behavior used to enact it. Yue and Ng (1999) obtained a similar result with the finding that old and young people alike held strong beliefs about filial piety, with obedience rated as the least filial concern, and respect as the highest. They concluded that there “appears to be a new cultural protocol for fulfilling filial obligations” (p. 215).

Yeh (1999) applied a historical approach. He examined how filial piety had evolved over time and identified stages of conceptual development linked to sociopolitical conditions that matched the authoritarian and affective elements Sung (1990) had identified. In the Pre-Chin era (551 to 221 BC), the fundamental concepts underlying filial piety were affection and the reciprocal nature of virtue. In other words, obligations were understood to be reciprocal (generosity should be returned in kind), and not based on hierarchy. Subsequently, from the Han to the Qing Dynasty (206 BC to 1911), the idea that inferiors must submit to superiors became more prominent, filial requirements became stricter, and violation required severe punishment.

Yeh (2003) argued that restricting conceptualization of filial piety to “the choice of giving it up or keeping it” cannot address the complexity of the concept (p. 79). He proposed that with modern social, political, and economic development, “a new type of filial piety that is adapted to societal changes” may be emerging in which “some attributes of filial piety are gradually eroding at a conceptual level, although other attributes are still crucial to people’s everyday lives” (p. 71). For example, in Taiwan, the importance of the passive obedience and submissive aspects of filial piety were decreasing, but the active affective aspect of filial piety encompassing care for parents was strengthening (Yeh, 1997).

A content analysis of items based on 15 historical dimensions of filial piety (Yang et al., 1989) revealed four filial piety factors (Respect and Love parents, Support and Memorialize Parents, Oppress Oneself, and Glorify Parents). From these, Yeh (1997) identified two higher order factors that corresponded to the two stages of historical development of filial piety: reciprocity and authoritarianism. Yeh (2003) proposed the DFPM, and Yeh and Bedford (2003) validated it with the dual filial piety scale (DFPS), which they applied to investigate the correlations of the reciprocal and authoritarian factors with personality traits, attitudes, behaviors, cognitions, and affect.

Yeh and Bedford (2003) found that authoritarian filial piety (AFP) had a significant positive correlation with Neuroticism, and significant negative correlation with Openness, just as Zhang and Bond (1998) had previously found using Ho’s (1994) filial piety scale. However, Yeh and Bedford also found that reciprocal filial piety (RFP) had the opposite relation with these two personality traits. RFP had a positive correlation with Openness and a negative correlation with Neuroticism. These results suggest that definition of filial piety solely in terms of authoritarian norms/practices/values misses the full picture. Instead, the two dynamic aspects of filial piety must be considered together to obtain a complete understanding of the role of filial piety in modern Chinese societies.

In sum, early studies of filial piety conducted in Confucian societies that focused on authoritarian/hierarchical aspects described filial piety as unidimensional and focused on sacrifice and obedience. Those scholars who took other approaches (philosophical, phenomenological, or historical) found filial piety to be multidimensional with the addition of an affective/relational dimension. Interestingly, the study of filial piety in non-Confucian societies has followed a similar trajectory in which the initial focus on authoritarian aspects of filial piety expanded to include consideration of affection. We review two examples in the following section.

## Examples of the Conceptualization and Measurement of Filial Piety in Non-Confucian Societies

Many scholars have pointed out that parents in most societies expect a degree of filial piety from their adult children and that most adult children recognize care for their parents as a duty (Peek et al., 1998; van der Pas et al., 2005). In fact, *filial piety* first became a scholarly concept in the analysis of Western societies and Sinologists borrowed the term to apply to similar aspects of Chinese society (Hamilton, 1990). Examining the origins of the term *filial piety* provides insight into how Westerners tend to conceptualize the term.

*Filial* and *piety* derive from Latin; *filius* refers to being a son in relation to a parent, and *pietas* means dutiful (Hamilton, 1990). Hamilton noted that Roman Law emphasized the power of the father, “which implies duty and obedience on the part of the son” (p. 84). This point is an important difference from the Chinese conceptualization of filial piety, which emphasizes the duty and obedience of the son, and only implies the power of the father. In the original conceptualization under Roman Law, the father’s power required filial piety as a duty on the part of the son with an inherent obligation to sacrifice, support, or obey. From this perspective, affection is not considered part of filial piety. Scholars have noted the influence of Western perspectives, theories and models in the research of Chinese academics when they return home from study abroad (Yang, 1997). It may be that the earliest psychological researchers in Chinese societies (who assumed a unidimensional authoritarian perspective on filial piety) were influenced by this Western definition of the term.

In the following, we review two broad frameworks that have been applied to investigate the role of filial piety in relation to elder care in various societies: the intergenerational solidarity framework and familism. As with early Chinese filial piety studies, research in both these areas tends to define filial piety in terms of social norms.

### Intergenerational Solidarity: Filial Expectations and Obligations

A prominent model that has been applied to study the role of filial norms in adult children’s support of aging parents emphasizes duty and obligation and excludes affection. The intergenerational solidarity framework (Bengtson and Roberts, 1991) defines *filial obligation* as part of normative solidarity: “the strength of commitment to performance of familial roles and to meeting familial obligations (familism)” (p. 857). In this model, helping/support behavior, reciprocity, association, and affection are seen as independent dimensions from filial obligation. Studies applying this model generally measure filial norms with four items in which participants rate the strength of filial obligations pertaining to the requirement for children to maintain geographical proximity/live with their parents and their willingness to make sacrifices in order to support their parents (e.g., Lee et al., 1994; Lowenstein and Daatland, 2006). These elements reflect aspects of AFP as conceptualized by Ho (1994, 1996) in his conceptualization of Chinese filial piety.

Early studies applying this model defined filial norms in terms of aging parents’ filial expectations, perhaps because of the inherent emphasis on parental power embedded in the concept. These studies did not find a clear relation between filial expectations and the amount of support that adult children provided (e.g., Lee et al., 1994; Eggebeen and Davey, 1998), including long term studies (e.g., Peek et al., 1998) and more recent studies with participants from other societies (India-Diwan et al., 2011; e.g., Dominican Republic/Puerto Rico, Boucher, 2017).

Later studies measured adult children’s sense of filial obligation to their aged parents (e.g., Lowenstein and Daatland, 2006; Silverstein et al., 2006; Dykstra and Fokkema, 2012) and did find evidence of a predictive relationship between the adult children’s feeling of obligation and the amount of support provided. For example, Lowenstein and Daatland (2006) found that the effect of filial norms on the level of support provided by adult children was significant across five European countries. A similar result with the intergenerational solidarity model has been found in Asian societies. Lin and Yi (2013) operationalized filial norms narrowly as the adult children’s perceived obligation to provide financial support to parents and found that the more adult children in China, Japan, Korea and Taiwan endorsed filial norms, the more likely they were to provide support to elderly parents, irrespective of other relevant factors.

Similarly, studies of South African Muslims (Ramaboa and Fredericks, 2019) and Muslim immigrants to France from North Africa (Duguet et al., 2016) examined the filial obligation of adult children to provide support for their aging parents. Both studies cast filial piety as an integral aspect of Islam and found that greater endorsement of this filial obligation corresponded to the increased behavior of caring for an aging parent in the home. Both studies evoked modernization theory and expressed the expectation that this filial value (and therefore the behavior) would decrease with modernization and/or exposure to Western cultures.

In fact, studies applying the normative solidarity framework have demonstrated that religiosity has a positive significant correlation with endorsement of filial norms and parental support in numerous societies (Norway, England, Spain, Germany, Israel; Gans et al., 2009; Italy, Tosi and Oncini, 2020). These studies measured filial obligation in terms of endorsement of obedience norms (an aspect of AFP) and noted that traditionalism may underlie the connection between filial norms and religiosity. This perspective mirrors that of the early Chinese research on filial piety (e.g., Ho and Kang, 1984; Ho, 1996), which cast filial piety as a static set of beliefs or attitudes and practices anchored in traditional norms.

### Intergenerational Solidarity and Affection

Researchers have considered affection in relation to filial piety from a variety of perspectives. Some have argued that support of aging parents is motivated by affection *instead of* filial duty (Swartz, 2009). Others asserted that affection is a motivator of filial obligation, which motivates support (Finley et al., 1988), although it is not sufficient in and of itself (Jarrett, 1985). Bengtson and Roberts (1991) recognized that family relationships encompass normative expectations for emotions as well as support. They found that normative solidarity (their umbrella concept for filial obligation) strongly predicts affection in their intergenerational solidarity model.

Silverstein and Bengtson (1997) refined the intergenerational solidarity model with two general domains of intergenerational cohesion: latent and manifest solidarity. Latent solidarity encompasses feelings of obligation and emotional closeness, and manifest solidarity represents functional exchanges of emotional, instrumental, and material support. The latent factors enable the functional support to emerge.

Latent solidarity encompasses the two intertwined aspects of filial piety identified in Chinese research with the DFPM (i.e., Yeh and Bedford, 2003, 2004): the external social obligations that exert pressure to conform in order to fit in with the wider social expectation (AFP), and the nature of the relationship with the parents (RFP). However, researchers applying the intergenerational model have tended only to investigate the relation between the former (obligation) and the instrumental and material forms of manifest support (e.g., Lee et al., 1994; Peek et al., 1998), and only a few researchers have included affective/emotional aspects of manifest support. For example, van der Pas et al. (2005) applied two different methodologies to verify the importance of different types of manifest support in their study of elderly Dutch citizens (emotional, instrumental, material, and informational). Hamon and Blieszner (1990) focused on filial role enactment (manifest support) and unexpectedly found emotional aspects of the relationship to be important. They concluded that the nature of filial responsibility has changed. They found that older parents have higher emotional-oriented filial expectations than instrumental filial expectations, and that adult children tend to interpret the filial role as one that includes a high level of emotional support. They indicated that instrumental assistance was less important than in the past, and that affection and emotional support and advice were more important. These findings are similar to the second wave of Chinese filial piety research that encompassed both affect and obligation and concluded that filial beliefs may be changing with greater emphasis on the quality of the parent-child relationship (e.g., Cheung et al., 1994; Yue and Ng, 1999).

### Familism

*Familism* is prioritizing one’s family over oneself (Schwartz, 2007). It is defined as “a cultural value that involves…strong feelings of loyalty, reciprocity, and solidarity among members of the same family (p. 314).” Familism is generally associated with Hispanic culture, although it has been demonstrated to operate similarly in diverse ethnic groups in the United States (Schwartz, 2007). Scholars investigating Hispanic familism have noted a conceptual overlap with Chinese filial piety and hypothesized that they comprise a multicultural or transcultural dimension. Using large multi-ethnic samples, Unger et al. (2002) found that these two concepts are highly intercorrelated, and Schwartz et al. (2010) found that familism and filial piety combined to form a single factor of Family Primacy in a study including Asian, Middle Eastern, Hispanic, Caribbean, and Central and South American participants. Similarly, Lin (2018) found a correspondence between familism and filial piety in a sample of Hispanic, Asian, and Pacific-Islander students using measures focused on authoritarian aspects of obedience and submission to the family’s needs.

Schwartz et al. (2010) found that Family Primacy is “similar to vertical collectivism in that parents must be obeyed without question, and in that individuals must prioritize family members’ wishes and needs over their own” (p. 554). They also pointed out that endorsing this factor does not in any way counter American values or a sense of American identity; it is fully compatible with it. In other words, filial piety and familism are concepts that are relevant to all ethnicities, not just the originating ones. It is worth noting that Unger et al. (2002) defined and measured filial piety relying solely on the work of Ho (1994), who investigated only hierarchical authoritarian Chinese filial characteristics, and whose measure contained no items pertaining to affection. Schwartz et al. (2010) used Unger et al.’s measure of filial piety for their study. Thus, the overlap of familism and Chinese filial piety identified in these studies consists solely of AFP aspects.

Kao and Travis’ (2005) not only recognized an overlap between Chinese filial piety and Hispanic familism, they equated it with the Spanish concept of *familismo*, which they defined as the attachment and loyalty of individuals to their families consisting of: obedience to authority figures, helpfulness among family members, responsibility and sacrifice for family, and obligation to provide material and emotional support. They translated a filial piety measure developed in Chinese in Taiwan to develop the Expectations of Filial Piety Scale (EFPS), which was the first filial piety measure for Hispanic populations. Kao and Travis did not specify the authors of this Chinese measure, but we suspect it was based on the work of Yang et al. (1989). Similar to Yang et al. (1989), they identified four factors, which they labeled: Respect for Parents, Honoring Parents, Supporting Parents, and Family Unity. The first three factors are the same as the Chinese version and they relabeled the fourth factor Family Unity instead of Oppress Oneself. It is informative to compare the results of Kao and Travis’ (2005) factor analysis of Chinese filial piety items with a factor analysis of items used to develop a Hispanic familism scale (Steidel and Contreras, 2003). That analysis also identified four factors: Familial Honor, Familial Interconnectedness, Familial Support, and Subjugation of Self for Family. It is clear both familism and filial piety encompass elements of unity/loyalty, honor, respect, obedience, and the inherent expectation of support, especially in times of need. Additional evidence for including an affective aspect in the conceptualization of familism is provided by a qualitative study of Hispanic elders that concluded that affectual solidarity is a cornerstone of *familismo*, and that affection explains why elders reported high family support despite infrequent contact with family members (Ruiz, 2007).

Similar results have been obtained in Arab-Israeli culture. To develop the first filial piety scale in Arabic, Khalaila (2010) used some items from Kao and Travis’ (2005) Hispanic filial piety measure (consisting entirely of items translated directly from a Chinese filial piety measure), and developed some new items reflecting the motives and content of the local context. Khalaila applied modernization theory with the perspective that filial piety represents traditional norms, so she added new items that would negatively correlate with modernization and positively with religiosity. She identified seven dimensions: Sacrifice of Money and Time, Obligation and Affection, Respect for Parents, Culture and Family Face-saving, Desire to Repay, Intergenerational Exchange, and Family Unity and Harmony. She reported a relatively high level of filial piety among adult children in Arab-Israeli society.

## Problems With Conceptualizing Filial Piety in Terms of Behavior and Attitudes to Norms

The majority of studies reviewed in the preceding sections examined filial piety in terms of attitudes toward norms or normative behavior. Many conceptualized filial norms in terms of traditional values (e.g., Kao and Travis’, 2005; Khalaila, 2010). There are two main (interrelated) problems with defining filial piety in this way: identifying the norm/beliefs/attitudes for inclusion and recognizing when they change, and the cross-cultural applicability (including the implicit metaphysical stance) of this research approach. We discuss each in the following.

### Identification of Content and Evolution of the Concept

The first challenge is to identify which norms to select for conceptualization of filial piety. The norms selected for focus dictate the findings, and even result in opposing conclusions. For example, Ho and Lee (1974) conceptualized filial piety in terms of the structural norms of Confucian ethics relating to hierarchy. The 22-item scale they developed (Ho and Lee, 1974; Ho and Kang, 1984) contains 21 items with content related to submission, sacrifice, and obedience. One item mentions respect for parents. No items mention affection or caring. They concluded that filial piety was waning with modernization, a point of view that can still be found in more recent studies adopting this type of definition (e.g., Cheung and Kwan, 2009; Khalaila and Litwin, 2012). In contrast, Yeh and Bedford (2003, 2004) conceptualized filial piety in terms of both authoritarian and reciprocal norms. Their measure reflected two aspects of filial piety, with eight items targeting authoritarian elements, and eight items targeting reciprocal elements of parent-child interaction. They concluded that although some authoritarian aspects of filial piety are of less relevance, RFP remains a strong consideration in modern Confucian societies. Other scholars who focused on or included affective norms reached a similar conclusion (e.g., Cheung et al., 1994; Sung, 1995). Similarly, Unger et al. (2002) and Schwartz et al. (2010) identified familism with authoritarian aspects of filial piety, but Kao and Travis’ (2005) expanded their conception of familism to encompass both authoritarian and relational aspects. The measures applied in their respective studies reflect this difference in conceptualization.

Even if a consensus can be achieved with regard to the content of filial norms, using them to define filial piety implies either that it is necessary to measure an ever-widening gap with a static set of beliefs (and the impact of that gap on intergenerational relationships and personal growth), or that the definition of filial piety would need to continue to evolve with social change. A study in the United States analyzed the filial attitudes and beliefs of three generations of women and found that although all three generations had a strong sense of filial responsibility, the content differed between generations (Brody et al., 1983). Filial norms not only differ by generation, they may change for a given individual with personal circumstances, or even aging (Gans and Silverstein, 2006). Indeed, some researchers have already observed that it may be necessary to update the concept of filial piety for modern societies. Lum et al. (2016) developed a new conception and measurement for filial piety “in the 21st century” (p. 1235). Choi (2004) presented an argument for a “new concept of filial piety in modern Korean society” (p. 72). Fu et al. (2020) asserted that not only is it necessary to create a new measure because of new filial piety norms, it is also necessary to develop different measures for children, adult children, elderly parents, and the general public. This approach may create more questions than it resolves. For example: How does one know when to update the definition? How does one compare change over time if the definition also changes? Do different societies need different definitions?

### Application in Multiple Cultures

This last question raises another issue. If filial piety is defined in terms of cultural norms, the appropriateness of applying the concept to other cultures becomes an obvious question. It also raises a consideration about the very conceptualization of filial piety. In adopting a psychological approach to filial piety, early researchers relied on dispositional methods (Hwang, 2012). They defined filial piety in terms of Chinese norms and measured the strength of an individual’s attitudes to these norms in order to relate them to other psychological traits or behaviors. That is, they relied on the approach of mainstream psychology. Bedford and Yeh (2020) pointed out that the limitations of mainstream psychology are similar to the limitations identified in other natural science disciplines and highlighted the field of physics as an example. The subatomic realm could not be understood with the traditional scientific approach; a paradigm shift was needed to address wave-particle duality. The key to the paradigm shift was the recognition that interactions between a target and the environment can change the nature of the target. The paradigm shift allowed physicists to focus on the interactions between a target and its environment, rather than being restricted to a sole focus on a target and its properties or fundamental nature. From this perspective, it is not possible to understand a target without understanding its interaction with the environment. The implication for research in the field of psychology is that a person cannot be understood as an isolated autonomous being transacting with others. Instead, people must be understood as constituted by their relationships with others. For the study of filial piety, the significance is that an approach that puts the parent-child relationship at the center of investigation may yield different insights from approaches that focus on the attitudes and norms of individuals.

## Methodological Relationalism as an Alternative Paradigm

The paradigm shift in the natural sciences aligns with a body of research labeled *Chinese relationalism* that emerged with the Chinese indigenous psychology movement (Hwang, 2013). *Methodological relationalism* (Ho, 1991) is an alternative approach to methodological individualism for conducting research in psychology. From this perspective, because the relational context supplies important determinants of social behavior, examining individual variables alone is insufficient to predict individual behavior. Thus, the target of research investigation should not be the *individual*, but the *individual-in-relations*. Chinese metaphysical and philosophical traditions are aligned with this perspective (Bedford and Yeh, 2020). Chinese filial piety is not simply children’s respect and obedience or even care and love for their parents. Filial piety is grounded in the unity of heaven and humanity, so the practice of filial piety is not an individual act, but rather a communal act that joins the individual with his or her community *via* a shared worldview (Tu, 1985).

Yang et al. (1990) described the difference in the methodological individualism and methodological relationalism approaches with respect to the investigation of filial piety as follows: The mainstream psychological approach focuses on (1) the phenotypic expression or epiphenomenal manifestation of filial piety; (2) the content of social attitudes and behavior; (3) static dimensions; and (4) the relation among cognitive, affective, intentional, and action levels. In contrast, a relational approach focuses on (1) the genotypic foundation and phenomenological meaning of filial piety; (2) deep structure (the relations among elements rather than the element itself); (3) dynamics and diachronic dimensions; and (4) the process of transformation. In other words, the mainstream approach sees filial piety as equivalent to the individual’s behavior instead of as a social action. The mainstream approach horizontally targets phenotypic manifestations and does not dig down vertically to the foundation of filial piety at a genotypic level. Whereas the mainstream approach is only able to address the superficial filial behavior, a relational approach can investigate the deep structure of filial action. It is the difference between viewing filial piety as a skin or as a skeleton.

What we emphasize in this paper is that filial piety is better understood as a skeleton than as a skin. Instead of focusing on the attitudes and beliefs of individuals (skin or surface content), the focus can be shifted to identifying the underlying psychological principles or mechanisms (skeleton or deep structure) that connect individuals with their environment in the context of the parent-child relationship.

## Filial Piety as a Contextualized Personality Construct

Yeh (2009) recognized these limitations of defining filial piety in terms of cultural norms so he re-conceptualized the DFPM by shifting the focus of investigation from cultural norms to the inherent structure of the parent-child relationship, which encompasses both horizontal and vertical aspects. Each aspect reflects an underlying psychological motivation that guides children’s interactions with their parents (see Table 1). The horizontal aspect is reflected by RFP. It is motivated by genuine affection developed through intimate parent-child interaction and grounded in the equal relationship of two individuals. It meets the individual’s need for mutual relatedness. The vertical aspect is reflected in AFP. It is motivated by the child’s normative desire to satisfy parental demands and grounded in the asymmetry of the parent-child relationship. It meets the individual’s need for collective identification. Together, AFP and RFP provide a structure that connects these two underlying universal psychological needs at the individual level to the surface content of filial norms at the collective level. In this new conceptualization, the DFPM focuses on the dual mechanisms underlying parent-child relations which are expected to be universal, and not on filial norms, thus the model has the potential for application in any cultural context.

**TABLE 1:** The dual filial piety model: Psychological schemas for interaction with parents.

	Reciprocal filial dimension	Authoritarian filial dimension
Psychological needs	Need for interpersonal relatedness with another individual.	Need for collective identity with society or generalized others.
Manifestation in developmental stages	Infancy to adolescence: Emotional safety and affective bonding with parents through expression of love or affection. Adulthood: Strengthen affection and bonding with parents; understand and support parents’ life needs.	Infancy to adolescence: Avoid punishment and gain social reward (e.g., parental praise) by learning to obey parental demands. Adulthood: Enact the social role of child according to common behavioral standards.
Features of psychological functioning	Simultaneously satisfy mutual needs for relatedness and emotional safety of parent and child.	Consider others’ needs (parents, spouse, or whole family) prior to personal needs.
Structural features inherent in the parent-child relationship	Equal status between two individuals. Horizontal relations. Need fulfillment is based on individual traits or differences.	Unequal status between the different roles within the family hierarchy. Vertical relations. Need fulfillment is based on specific role norms.
Ethical principle of Confucianism	*qin qin*: Principle of favoring the intimate.	*zun zun*: Principle of respecting the superior.

*Adapted from Tsao and Yeh (2019).*

To extend this point, Bedford and Yeh (2019) applied the concept of *contextualized personality*. Whereas in the classical psychometric theory or trait approach, personality is typically conceived of in terms of an individual’s stable traits, with a contextualized approach, personality is understood to arise from the individual’s interaction with the environment. *Contextualized personality* is defined as the “stable patterns of thought, feelings, and behaviors that occur repeatedly within a given context” (Heller et al., 2007, p. 1229).

The contextualized approach differs in two important ways from the trait approach to personality. First, instead of conceptualizing personality traits as “causal forces used to predict outcomes that are not themselves subject to change” (Roberts, 2006), personality traits are understood to have the potential to manifest in different ways across “environmental contingencies often contained within social roles” (Roberts and Caspi, 2003). Second, personality is not defined solely in terms of traits; some aspects of personality are not represented by traits at all (Dunlop, 2015). In addition to traits, personality can also be expressed in terms of motivations/needs.

A *personality motivation* is a person-in-context variable—it cannot be separated from the context in which it is practiced. Social roles provide the context for defining personality motivations. In fact, personality motivations are distinguished from traits by their contextualized nature (McAdams, 2013). The social roles of the parent-child relationship provide the context for defining personality motivations related to filial piety.

Research suggests that social roles can be organized around two fundamental dimensions: belongingness/affiliation and status/power (Roberts, 2006). These dimensions allow people to satisfy the fundamental human needs of interpersonal relatedness (through intimate connections with others) and social belonging (by satisfying the normative expectations of the group/society). According to the reconceptualized DFPM, the parent-child social roles contextualize these two basic needs through RFP (interpersonal relatedness) and AFP (social belonging). In other words, the parent-child relationship provides the context for psychological conceptualization of filial relations, instead of using culturally-shaped behavioral norms as a basis for understanding filial relations (Bedford and Yeh, 2019). Because this approach focuses first on the context, the parent-child relationship (a universal context), and not on cultural content, it is appropriate for application in any culture.

Bedford and Yeh (2019) proposed that as a contextualized personality construct, the DFPM can facilitate four levels of analysis of filial relations: individual filial motivations (AFP and RFP); structural aspects of the parent-child relationship (vertical and horizontal); social changes related to filial norms; and differences across societies or cultures in individual motivations, relational structure, and filial behaviors (see Table 2). In the following, we provide examples of the application of the model by focusing on the individual level (although the levels are interrelated, so we touch on the others). Most studies applying the model have been conducted in Chinese societies. The model is intended to have universal application, so we also provide examples of how the DFPM may inform or integrate with filial research in other societies.

**TABLE 2:** Theoretical implications of the DFPM at different levels of analysis.

Level of Analysis	Corresponding implications	
	RFP	AFP
Basic psychological needs	Interpersonal relatedness	Social belonging and collective identity
Structural properties of parent-child relationship	Horizontal relationship between two unique individuals	Vertical relationship based on the family role hierarchy
Historical or social change	Core aspect of filial piety [relatively free from the impact of social change]	Changing aspect of filial piety
Cross-cultural comparison	Psychological prototype of filial piety	Cultural prototype of filial piety

*RFP, reciprocal filial piety; AFP, authoritarian filial piety. Adapted from Bedford and Yeh (2019).*

## Application of the DFPM and Integration With Research in Other Societies

As a contextualized personality construct, filial piety develops from childhood. Both authoritarian and reciprocal motivations are rooted in the intimacy and the quality of the parent-child relationship and have enduring influence on psychosocial adjustment as well as the parent-child relationship. In this section, we review research applying the DFPM to investigate psychosocial adjustment at the individual level of analysis, and to examine the parent-child relationship at the individual and structural levels of analysis.

### Psychosocial Adjustment: Individual Level of Analysis

A number of studies have applied a measure based on the DFPM, the dual filial piety scale (DFPS; Yeh and Bedford, 2003) to examine the authoritarian and reciprocal filial motivations in relation to various aspects of the psychosocial adjustment of adolescents. For example, RFP is positively correlated with life satisfaction (Wong et al., 2010; Sun et al., 2019) as well as good interpersonal skills (e.g., self-disclosure and empathy), reduced parent-child conflict (Yeh and Bedford, 2004), and better overall psychosocial adjustment (Yeh and Yang, 2006). RFP mediates the influence of supportive parenting on young adults’ happiness (Chen et al., 2016). RFP also corresponds to reduced depression and anxiety (internalizing) behavior and reduced deviant and aggressive (externalizing) behavior (Yeh, 2006) in Taiwanese adolescents. AFP reflects internalized role obligations to be comply with social norms. Research on junior high students and their parents in Taiwan showed that AFP beliefs are positively associated with traditional and conservative attitudes (e.g., male superiority and submission to authority; Yeh and Bedford, 2003) and maladaptation (e.g., neurotic personality traits, depression, and anxiety; Yeh, 2006). Because AFP often involves self-suppression, it is more likely to correlate with personal stress than RFP (Yang and Yeh, 2006), and it has a negative relation to self-esteem (Wong et al., 2010). A recent study demonstrated that although AFP has a negative indirect effect on life satisfaction through the mediating role of individuating autonomy, it has a positive indirect effect through relating autonomy (Sun et al., 2019).

An additional benefit of the DFPM is that rather than simply designating people as filial or un-filial, the model allows for a more nuanced examination of the psychological mechanisms underlying the affective reactions and social behavior associated with filial piety by identifying four possible modes of personal interaction with parents: high reciprocal-low authoritarian, high authoritarian-low reciprocal, high on both (balanced), and low on both (non-filial) (Yeh and Bedford, 2004).

These filial types can be applied to the investigation of topics that have been little considered in relation to filial piety such as attachment style (Ainsworth and Bell, 1970). The DFPM model may provide a strong tool for research in this domain since it encompasses and extends several aspects of attachment theory (Bedford and Yeh, 2019): The balanced type corresponds to secure attachment, the non-filial type corresponds to avoidant attachment, and the reciprocal and authoritarian types correspond to ambivalent attachment.

Rather than focusing solely on early childhood as the critical period of development as in attachment theory, the DFPM encompasses the entire lifespan and recognizes the relevance of the parent-child relationship as a person transitions to adulthood and beyond. It can apply to research investigating adult relations with parents. For example, the concept of *intergenerational ambivalence*, a mix of positive and negative feelings toward parents (Luescher and Pillemer, 1998), may be interpreted as an imbalance between RFP and AFP. Adolescent adaptation, social development, and delinquency are predicted by *parental authority* (Darling et al., 2008), which is similar to AFP in its focus on the vertical aspect of the parent-child relationship.

Application of the DFPM may also provide insight on the relationship between Hispanic familism and adolescent adjustment. Strong familism can shield against damage from the negative aspects of risk (Deane et al., 2020). Aspects of familism related to interconnection and pride serve as a protective factor for positive psychosocial outcomes (Stein et al., 2019). Familism may also moderate the relation between stress and internalizing and externalizing behavior under some conditions (Umaña-Taylor et al., 2011), although it has also been shown to relate to higher levels of internalizing behaviors (Kuhlberg et al., 2010), and to increase risk of distress among adolescents under some conditions (Hernández et al., 2010). From the perspective of the DFPM, the explanation for these inconsistent results may lie in conceptualization of the measure and whether it emphasizes AFP or RFP aspects of familism.

### The Parent-Child Relationship: Individual and Social Structural Levels of Analysis

The most prominent focus of filial piety research is elder care. Both RFP and AFP have the same functions of sustaining family solidarity and responsibility for elder care at societal level, but the underlying mechanism of each aspect is distinctive at the individual level (Yeh et al., 2013). Specifically, RFP facilitates intergenerational support for parents through authentic gratitude and affection; AFP does it through the motivation to meet social expectations. The behavior may be the same, but the filial motivation is different. The implications of this difference for both the adult child and the parent are many.

In Chinese societies, RFP not only correlates with the caregivers’ (adult children’s) reduced burden and stress, but also with the elderly parents’ enhanced self-worth (Chan et al., 2012). In addition, RFP correlates with adult children’s intention to remain as caregivers of their parents as well as the quality of care they provide (Hsiao and Chiou, 2011). Neither of these studies found these positive benefits in relation to care attributed to AFP. RFP has been shown to have a significant positive relation with the frequency of assistance with household chores, financial support, as well as emotional support of aging parents. In contrast, AFP only showed a positive relation with the frequency of help with household chores (Yeh, 2009). This finding is similar to the results of studies applying the intergenerational solidarity theory that defined filial piety in terms of requirements to live near parents and make sacrifices (AFP aspects). As Gans et al. (2009) noted, the majority of these studies found only a weak relationship between norms and support behavior (e.g., Silverstein et al., 2006).

Many societies face issues related to intergenerational support. A study based on a representative sample of Africans, Latinos, Asians, and Europeans concluded that there may be more similarities than differences in beliefs about intergenerational assistance (Coleman et al., 2006). The DFPM may be applied as a framework for investigation of this context. For example, in South Africa, the term *black tax* refers to financial transfers from black middle class individuals to their families, usually in rural areas (Mangoma and Wilson-Prangley, 2019). These remittances have been examined in depth from an economic perspective; there are fewer studies from an ethnographic perspective, and no dominant ethnographic approach (Carling, 2014). For the individual making the transfer, a narrow focus on economic aspects highlights the frame of a “tax” or a penalty in which these professionals are always encumbered with remittances to family members instead of investing or saving for retirement. They cannot get ahead in life. In contrast, a qualitative study explored the perspective that the remittances can be a bond that ties individuals to their families. Mangoma and Wilson-Prangley (2019) found that most participants had mixed feelings encompassing both obligation and gratitude for sacrifices that family members had made for them, and they recognized that these family sacrifices had given them opportunities and allowed them to succeed. Some participants described the payments to family members as “lightening their load” rather than paying them back (p. 11). The majority expressed that despite any negative impact on their own success, “there are benefits to the transfers and that the experience is beneficial” in terms of a sense of satisfaction and strengthening intergenerational relationships” (p. 13). Remittances may be material, but they can also be relational and emotional. Whereas the economic perspective focused on social norms and obligation (AFP aspects), the ethnographic approach identified gratitude and personal relationships (RFP aspects) as the key to understanding the dynamics at work in this context. Application of the DFPM as a framework for investigating this context would suggest that it is important to consider both together.

## Measuring Filial Piety

We began our review of filial piety research with a description of the earliest measure of filial piety, which was developed in Hong Kong (Ho and Lee, 1974). That measure suggested only authoritarian aspects of filial piety are relevant. To bring this review full circle, we conclude by mentioning a recent study conducted in Hong Kong that proposed a new measure of filial piety. The authors made the broad claim that “filial piety is no longer perceived as an authoritative obligation” and proposed a measure in which they attempted to remove the authoritarian aspect and focus solely on the relational emotional aspect of filial piety in the context of support for aging parents (Lum et al., 2016).

In this section, we review Lum et al.’s measure along with two other recently proposed measures of filial piety (i.e., Shi and Wang, 2019; Fu et al., 2020). These studies illustrate the problem of defining filial piety in terms of norms, as well as the importance of understanding the history of the conceptualization of filial piety and the content of previous measures.

### The Relevance of Authoritarian Filial Piety

In the previous section, we described a body of research on elder care in Chinese societies indicating the positive benefits of RFP that were not similarly present with AFP. Given all those findings, it is easy to comprehend why one group of researchers advocated eliminating AFP from the measurement of filial piety altogether (i.e., Lum et al., 2016). Their argument was that filial norms have changed due to modernization and social change and thus there is a need for an updated measure. Grounding the definition of filial piety in social norms leads to this kind of reasoning. Although it may seem that according to the literature on elder care from Chinese societies, RFP provides all the benefits and AFP contributes little or causes problems, in the Confucian view, the ultimate goal is balance and harmony. The two motivations are intertwined, and both are necessary for human functioning. We thus contend that the authoritarian aspect is essential to understanding filial piety and that disregarding it would be a mistake for two main reasons.

First, even if AFP is waning in Chinese societies as a motivator for elder care, the concept may still be relevant in other societies in this context. Both aspects of filial piety were identified as relevant to filial measures developed for Hispanic (Kao and Travis’, 2005), Arabic (Khalaila, 2010), and Malay (Tan et al., 2019) societies. Also, researchers currently apply concepts related to AFP (only) to investigate filial obligation to support parents. For example, *moral capital* is the stock of internalized social norms regarding children’s obligation to care for their older parents (Silverstein et al., 2012). The researchers developed this term for application in conditions in which children’s gratitude or emotional bonds with their parents are insufficient to explain support of their parents. They argued that moral capital (an aspect of AFP) guarantees children’s support of aging parents even with a weak parent-child relationship.

Second, both authoritarian and reciprocal filial motivations can be used to understand intergenerational relationships beyond just care for aging parents. We described previously some research findings that applied the DFPM to investigate adolescent development; AFP and RFP are suitable for investigating parent-child relations throughout the lifespan. Although AFP and RFP are conceptualized separately for analysis, they are often intertwined in important ways, which allows a highly nuanced understanding of complex conditions. We provide three examples of the application of the DFPM to parent-child relations at different levels of analysis to illustrate this point.

Until the 1990s, some young girls in Taiwan from impoverished families were indentured by their parents to brothels, usually to cover family medical or gambling debt (Hwang and Bedford, 2003). Parents appealed to their daughter’s sense of filial piety to obtain her consent to indenture. Although they had little choice, the girls reported that they had not been forced; they had willingly agreed (an AFP-motivated decision). These girls were admired by their home communities for their sacrifice to support their family, and also for the income they provided. Filial prostitutes saw themselves as superior to the runaway prostituted girls and would not socialize with them in their free time. The filial girls had a much lower incidence of drug and alcohol abuse than the other girls (Hwang and Bedford, 2004). These girls had internalized their parents’ values and felt they were demonstrating their love for their parents by supporting them (an RFP motivation), illustrating the complex intertwined nature of the two filial motivations.

The second example is from a recent qualitative study, which found that some single women in Taiwan purposefully choose careers requiring a lot of overtime and socializing outside of the office in order to deflect parental pressure to start a family (Bedford, 2016). Many had concerns about obligations to in-laws (with whom they would have an AFP relationship, at least in the beginning). They also did not want to directly object to their own parents’ wishes. But, given their RFP relations with their own parents, they felt that their parents could accept a delay in marriage in order that their daughter experiences the job or career she likes. If women decide to delay or avoid marriage and family in order to escape the AFP obligations of in-laws, it could contribute to Taiwan’s low birthrate, which would be an example of filial piety contributing to social trends, instead of the usual assumption that social trends influence filial beliefs.

The third example is that both aspects of filial piety have been used as political tools in China. To consolidate their power, imperial rulers stressed authoritarian moralism (AFP). Communist leaders later worked to eliminate filial beliefs in order to promote the centrality of the state (An, 2009). China’s authorities switched tactics again to focus on reciprocal aspects of filial piety as a means of addressing the oncoming elder-care crisis with population aging (Xu, 2001). The 1980 Marriage Law gave parents the right to require regular payments from their children (Qi, 2015), and the Family Support Agreement (FSA) is a voluntary (yet legally binding) contract between parents and their adult children detailing obligations of ongoing support. From the perspective of the DFPM, the Chinese authorities are applying authoritarian tactics in an effort to promote RFP, which is unlikely to obtain the desired result. Indeed, Chou (2011) found that the FSA eroded spontaneity, flexibility, and affection in families (all aspects of RFP), which damaged relationships and resulted in an increase in family lawsuits. This example illustrates the importance of understanding the two filial motivations in order to design effective government policy around elder care and population aging.

Lum et al.’s (2016) measure illustrates what can happen when filial piety is defined in terms of norms in a particular context in a particular culture at a particular point in time. Their measure may be able to assess the surface behavior related to filial piety in Hong Kong in the specific context of elder care. It is not able to address filial piety in general or speak to the deeper motivations underlying filial behavior.

### Normative Filial Piety in Three Dimensions

Like the authors of the measure just reviewed, Fu et al. (2020) highlighted “new filial piety norms” as the rationale for developing their measure (p. 177). Whereas Lum et al. (2016) developed a general measure applicable to all ages in Hong Kong, Fu et al. (2020) asserted that because the concept of filial piety relates to different generations, “different groups of people might have different understandings of and standards for what constitutes filial piety” (p. 180). Their aim was to create a new measure of filial piety for elders aged 70 or older with the expectation that “contemporary elders in China hold less authoritative but more reciprocal stances toward filial piety” (p. 176). Their study illustrates the importance of understanding the previous conceptualizations and content of earlier measures.

Fu et al. (2020) invoked research grounded in the intergenerational solidarity paradigm (Silverstein et al., 2002) to support their contention that the parent-child relationship (and thus filial piety) is reciprocal in nature. However, this research casts reciprocity as grounded in a cost-benefit analysis that is external to filial obligation. That is, filial obligation explains parental support in the absence of reciprocity. This stance does not accord with the author’s premise that elders expect reciprocal aspects of filial piety to motivate their children to support them.

To develop their measure, Fu et al. (2020) pooled 38 items from three measures (Ho, 1994: 22 items, Yeh et al., 2013: six items, and Lum et al., 2016: 10 items). Most of these items represent aspects of AFP. Their analysis identified three factors with a total of 12 items: Caring for Parents (six items: described as representing the practical and emotional aspects of reciprocity), Familial Entirety (three items: described as a new domain related to elders’ traditional Chinese normative values), and Familial Aspiration (three items: described as relating to authoritative obligation).

The authors concluded that their analysis indicated that elders in China are more reciprocal than authoritative in their stance toward filial piety. However, all the items in their measure start with the phrase, “Children should…,” which indicates a normative authoritarian orientation. Thus, the measure seems to address three domains of normative obligation: treatment of parents, carrying on the family name, and bringing honor to the family. It does not address reciprocity in the parent-child relationship.

In sum, Fu et al.’s scale is concerned with expected filial behavior, not filial motivation. It can assess the expectation, but it cannot discriminate among underlying motivations for performing that behavior as the authors claim. This scale sheds light on what Chinese elders think their children should do, but not why they think they should do it.

### Measuring Filial Piety Motivations With Three Dimensions

We have made the case for a shift from a surface (skin) focus on norms to a deep structure (skeleton) focus on the psychological motivations of filial piety. Shi and Wang (2019) recently made the case for focusing research on filial motivation with the argument that motivation is the central process for generating moral judgment and action, and proposed a new measure of filial piety, the three-dimensional filial piety model (TDFPM). Each of the three dimensions consists of two opposite poles: *good affection* (true–false), *family role norms* (autonomy–heteronomy), and *balance of interests* (reasonable–unreasonable). Shi and Wang explained that the fundamental difference in the two poles of each dimension lies in the individual’s motives. We agree and assert that in fact each of the three dimensions of the TDFPM can be expressed by casting the reciprocal and authoritarian motivations of the DFPM in opposition to one another.

That is, true affection, autonomous filial piety, and reasonable interests are grounded in RFP; false affection, heteronomous filial piety, and unreasonable interests are grounded in AFP. Whereas in the DFPM, AFP and RFP are independent dimensions, in the TDFPM, they are cast in opposition to one another and measured in conjunction with other concepts (instrumentality, self-awareness, and balanced interests). This configuration reduces the range of filial motivations that can be investigated since it does not allow for an individual to be high or low in both AFP and RFP.

The TDFPM focuses on motivations, which is a positive step that can allow for a more nuanced analysis of behavior than the traditional focus on filial norms. The model highlights some aspects such as instrumentality, self-awareness and balance of interests that may be useful to explore in the context of filial interaction. The model may be critiqued in that instead of adding new insight into the psychological motivations related to filial piety, it constricts the range of information available by integrating reciprocal and AFP with other concepts instead allowing for an investigation of the relationships among these and other concepts.

## Conclusion

We reviewed some of the early literature on filial piety conceptualization and measurement. Studies from a variety of societies have converged on an understanding of filial piety that is associated with authoritarian/normative and emotional/relational aspects, although scholars have differed in their assessment of the relation of affection to filial piety. Studies that focused on norms have tended to associate filial piety with traditional beliefs, which limits the cross-cultural applicability of subsequent measures, and implies the necessity of updating the measures as norms change.

Development of psychology as a global science capable of representing the full human experience requires the ability to incorporate both cross-cultural invariance and cultural variation. By viewing the dual aspects of filial piety as fundamental psychological motivations grounded in the parent-child context, the DFPM is able to meet both of these criteria. According to Norenzayan and Heine’s (2005) article conceptualizing psychological universals, a psychological phenomenon is a functional universal if the relationship between the variables remains the same, even if the strength of the pattern differs across societies. When filial piety is conceptualized as a contextualized personality construct, it meets this requirement.

Instead of focusing on the behaviors, or attitudes toward norms of individuals (skin or surface content), the focus is on understanding the underlying psychological motivations (skeleton or deep structure) that support behavior and connect individuals with their environment in the context of the parent-child relationship. In other words, a filial behavior may be motivated by affection or obligation, each of which has its own set of implications.

Concepts that focus on a universal context, such as parent-child interaction, can reflect local characteristics as well as shared elements of human behavior (Tsui, 2004). The DFPM connects the two universal individual filial motivations to the context in which they are most relevant. It highlights the horizontal and vertical aspects of parent-child relations that respectively reflect the reciprocal and authoritarian motivations that shape children’s interactions with their parents. The horizontal (RFP) is motivated by genuine affection developed through intimate parent-child interaction and grounded in the equal relationship of two individuals. It meets the individual’s need for mutual relatedness. The vertical aspect AFP is motivated by the child’s normative desire to satisfy parental demands and grounded in the asymmetry of the parent-child relationship. It meets the individual’s need for collective identification.

At the individual level, the DFPM is a tool for investigating the basic filial motivations of children and structural properties of the parent-child relationship. At the level of society, the DFPM can be applied to investigate social change or cross-cultural differences. At this level of analysis, the core aspect of filial relations, RFP, is relatively stable and is likely to share characteristics across cultures, whereas AFP is able to capture social change because it continues to reflect the internalized role obligations contained in cultural filial norms even though parents’ authority over their children diminishes as the children grow up. In other words, AFP reflects the schema for social belongingness and collective identity that is necessary to be seen as a member of a particular social or cultural group. Over time, these norms are likely to evolve with broader social changes.

These two aspects of parent-child interaction are entwined in daily life; we distinguish them at a theoretical level in order to comprehensively parse the patterns of parent-child interaction. As inherent universal structures, the horizontal and vertical relational aspects provide a solid base for comparing similarities and differences in the ideal for parent-child interaction pattern across cultures. Social change or cultural norms only influence the relative weightings of each filial motivation. No matter how the corresponding behavioral norms change (more strict or loose), these two filial aspects will still continue to exist and function psychologically.

## Data Availability Statement

The original contributions presented in the study are included in the article/supplementary material, further inquiries can be directed to the corresponding author/s.

## Author Contributions

OB and K-HY contributed to the article and approved the submitted version.

## Conflict of Interest

The authors declare that the research was conducted in the absence of any commercial or financial relationships that could be construed as a potential conflict of interest.
